# Mental State Attributions Mediate the Gaze Cueing Effect

**DOI:** 10.3390/vision2010011

**Published:** 2018-02-19

**Authors:** Emma J. Morgan, Megan Freeth, Daniel T. Smith

**Affiliations:** 1 Department of Psychology, Durham University, Durham DH1 3HP, UK; 2 Department of Psychology, University of Sheffield, Sheffield S1 2LT, UK

**Keywords:** mental states, theory of mind, gaze cueing, social attention

## Abstract

Understanding the mental states of our social partners allows us to successfully interact with the world around us. Mental state attributions are argued to underpin social attention, and have been shown to modulate attentional orienting to social cues. However, recent research has disputed this claim, arguing that this effect may arise as an unintentional side effect of study design, rather than through the involvement of mentalising processes. This study therefore aimed to establish whether the mediation of gaze cueing by mental state attributions generalises beyond the specific experimental paradigm used in previous research. The current study used a gaze cueing paradigm within a change detection task, and the gaze cue was manipulated such that participants were aware that the cue-agent was only able to ‘see’ in one condition. The results revealed that participants were influenced by the mental state of the cue-agent, and were significantly better at identifying if a change had occurred on valid trials when they believed the cue-agent could ‘see’. The computation of the cue-agent’s mental state therefore mediated the gaze cueing effect, demonstrating that the modulation of gaze cueing by mental state attributions generalises to other experimental paradigms.

## 1. Introduction

Our social interactions rely upon the capability to understand and track a social partner’s beliefs, desires, and intentions—an ability known as theory of mind (ToM) [1]. Through the act of taking another’s perspective, we ‘mentalise’ and compute to what and where another person is attending. Mental state attributions (MSAs) guide attention to subtle social cues and information that might otherwise go unheeded, thereby allowing us to successfully integrate with the social world around us. Indeed, one influential but controversial idea is that the ability to make mental state attributions underpins the phenomenon of social attention [2] and therefore occurs prior to gaze following. 

Social attention is often operationalized using a gaze-cueing task [3]. In the canonical version of this task, a participant is shown a face with the eyes averted, such that it appears to be looking at one side of the display. A stimulus is then presented on the side of the display the face is gazing at (a valid trial), or contralaterally to the direction of gaze (an invalid trial). The participant must quickly and accurately identify the target while maintaining fixation at the center of the screen. A robust finding is that reaction times are significantly faster on valid trials (i.e., when gaze direction is congruent with target location). The shift of social attention elicited in the gaze-cueing task is often characterized as a rapid, reflexive process [4], but the cognitive processes underlying this form of social attention appear to be dissociated from those involved in reflexive orienting to salient peripheral cues. For example, reflexive orienting is known to rely on the ability to program eye-movements [5,6], whereas social attention does not [7]. Indeed, several lines of evidence are typically cited in favour of an alternative, mental state attribution hypothesis of social attention. Both children [8] and adults [9] have been shown to differentially follow a gaze cue depending on whether its eyes are open or closed/masked, suggesting that the observers are accounting for the perspective of the cue in tasks that engage social attention. In a key study, Teufel and colleagues [2] directly manipulated a participant’s belief about the mental state of an agent. During a gaze cueing task, participants watched videos of an actor wearing one of two pairs of coloured goggles. Prior to the experiment, participants were informed that the actor could see through one of the pairs of goggles but not through the other. Teufel et al. carried out two experiments, the first with a non-predictive gaze cue and the second with a counter-predictive gaze cue. In the first experiment, there was a significantly larger gaze cueing effect when participants believed that the cue-agent could see. In the second experiment, participants were significantly less able to suppress reflexive gaze cueing when they believed that the cue-agent could see. In a related study, Wiese and colleagues manipulated observers’ beliefs about whether a cue-stimulus was being intentionally controlled, and observed greater gaze cueing effects when participants attributed intentional states to the cue [10]. Taken together, these results suggest that mental state attributions interacted with, and mediated the gaze cueing effect, thereby modulating attentional orienting in response to a social cue. 

However, more recent research has contested this interpretation, arguing instead that these studies confound mentalising processes with changes to the physical properties of the stimulus. In particular, masking the eyes of the stimulus has been argued to lead to the weaker gaze cueing effects found [11,12]. Indeed, in a series of experiments designed to explicitly test the mental state attribution account, Cole and colleagues [11] used a ‘line-of-sight’ manipulation in which barriers were placed between the cue-agent and the stimulus and either ‘opened’ or ‘closed’ to create the impression that the cue-agent could either see or not see through these barriers. Contrary to Teufel et al. [2], Cole et al. observed no effect of mental state attribution on gaze cueing. This null effect was even observed in vivo, when the cue was a real person gazing to left and right, leading Cole et al. to conclude that gaze cueing did not depend on a mental state attribution. Similarly, others have proposed that the apparent modulation of social attention by mental state attribution actually arises as a side effect of study design [13,14]. Together, these studies suggest that either gaze-cuing is driven by non-mentalistic processes, or if mentalistic processes do contribute, they only do so under very highly constrained experimental situations. 

Given the significance of Teufel et al.’s original finding, and Cole et al.’s subsequent claim that the effects of mental state attribution on gaze cueing did not generalise to other tasks, an important next step is to establish whether the ‘goggles’ effect generalises beyond the specific experimental paradigm reported by Teufel. The current study therefore aimed to replicate and modify the study reported by Teufel et al. [2]. Specifically, while our mental state manipulation followed the format of Teufel et al. [2] with the gaze cue pictured wearing one of two pairs of coloured sunglasses; one associated with ‘seeing’, the other with ‘non-seeing’, there were three methodological changes. 

First, following Cole and colleagues, we used a change detection paradigm [15,16] rather than a timed discrimination task. The effects reported by Teufel et al. were only present when they analysed the Inverse Efficiency and not the RT data. This analysis is potentially problematic, as IE is only appropriate when there is no speed-accuracy tradeoff. This may not have been the case in some conditions in Teufel et al. [2] (see Table 1, Long SOA condtion where participants are slower but more accurate in the Valid condition). It is therefore important to establish that the effect of mental state attribution can be observed using other measures of performance. Change detection tasks are more sensitive to behavioural changes [17] and less susceptible to noise [18] than response time tasks and highly sensitive to attention, such that participants are significantly more accurate at identifying changes that occur at a cued location [19,20,21], thus giving us the best possible chance of observing subtle modulations of cueing that may be lost in RT tasks. 

Second, we interleaved ‘seeing’ trials with ‘non-seeing’ trials, whereas Teufel et al. blocked their conditions. This allowed us to examine whether participants could flexibly update their mental state attributions on a trial-by-trial basis. This manipulation is an important modification of the previous work, as using a blocked design may have encouraged participants to adopt an attention control setting that suppressed orienting in response to the red glasses. For example, Folk, Remington, and Johnston [22] have shown that participants can suppress reflexive orienting to peripheral cues in blocks of trials in which the peripheral cue does not share physical properties with the target. Indeed, they showed that a red colour singleton failed to summon attention if the target was a luminance change. If participants can adopt an attentional set that suppresses covert orienting, one might argue that the results of Teufel et al. arise from this control setting rather than the effect of mental state attribution per se. Importantly, these attention set effects are very hard to implement when the participant does not know the identity of the cue at the start of the trial. 

Finally, participants were given no explicit instruction to adopt the mental state of the cue. This is important because it offers a stronger test of the idea that the mental state attribution occurs spontaneously and without requiring conscious effort by the participant to take the perspective of the cue-stimulus. It is also possible that the very act of asking participants to report on the mental state of the avatar makes the participant believe that mental states are important, and they are expected to behave differently in the ‘seeing’ and ‘non-seeing’ conditions. 

To briefly summarise, Teufel et al. [2] argue that social attention is mediated by mental state attribution. This idea has been highly influential, but we believe some caution is required before accepting this conclusion. First, the original analysis may violate some of the requirements for using Inverse Efficiency, secondly the use of a blocked design may allow participants to adopt attentional control settings that confound the effects of mental state attribution, and thirdly, explicitly instructing participants to consider mental states introduces a task demand that may influence the pattern of responses. To address these issues we adapted the Teufel et al. [2], paradigm such that participants were shown two brief videos, each approximately 15 s in length (Video S1: GlassesDemo.wmv). The videos were designed to instil the concept that the actor could ‘see’ whilst wearing one pair of coloured sunglasses and could not ‘see’ whilst wearing a differently coloured pair of sunglasses. Participants then performed a change detection task in which a cue-agent could be looking at the item that changed, or one of the distracters. Critically, trials where the cue-agent could ‘see’ and trials where he could not were randomly interleaved. In line with Teufel et al. [2]’s study, we predicted that participants’ gaze cueing would be mediated by the mental state of a cue-agent. We therefore expected that participants would be significantly more likely to detect if a change had occurred on the valid trials in the seeing, compared to the non-seeing, condition.

## 2. Materials and Methods

### 2.1. Participants

Thirty-four participants (24 female and 10 male), with a mean age of 24 years (range = 19–40, SD = 4.60) took part in the study. Undergraduate Psychology students received course credit for taking part. The study was approved by the Department of Psychology Ethics Committee, and all participants gave informed consent before participating. All participants had normal, or corrected to normal, vision. 

### 2.2. Design

The study used a within-subjects design with three independent variables: condition (seeing or non-seeing), validity (valid or invalid), and stimulus onset asynchrony (SOA) (180 ms or 1080 ms). The use of two SOAs allowed a measure of early processing and later top-down effects. The experimental trials were randomised across these three variables. The study paradigm was a change detection task, which required participants to correctly identify if one of four symbols (displayed in each corner of the screen) had changed.

### 2.3. Materials & Apparatus

**Figure 1:**
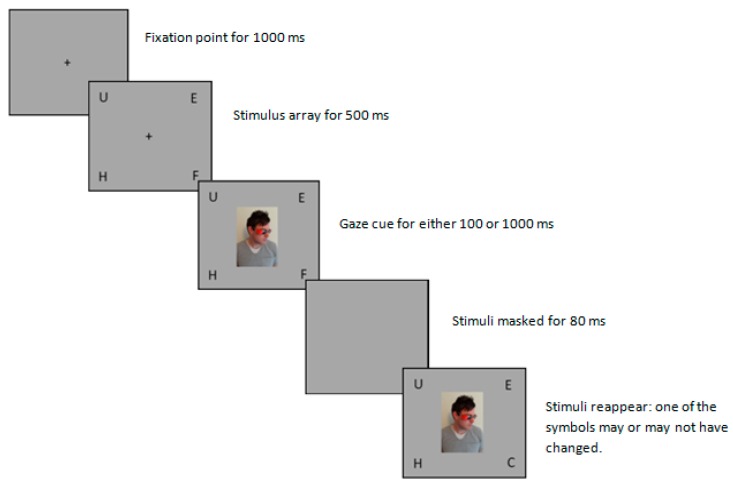
The experimental procedure. Trial types were randomised based on validity, condition, and stimulus onset asynchrony (SOA). The figure illustrates a valid trial.

All stimuli were generated using a ViSaGe graphics card (Cambridge Research Sytems Ltd, Kent, UK). Responses were collected using a keyboard. The paradigm used photographs of an actor wearing a pair of either red or yellow sunglasses. Participants were informed that the actor was only able to see whilst wearing one of the pairs of sunglasses, with the colour of the ‘seeing’ sunglasses counterbalanced between participants. Therefore, half of the participants were informed that the actor was able to ‘see’ whilst wearing the red sunglasses but was unable to ‘see’ whilst wearing the yellow sunglasses; the other half of the participants were informed that the actor was able to ‘see’ whilst wearing the yellow sunglasses but was unable to ‘see’ whilst wearing the red sunglasses. In total, there were eight photographs of the actor wearing each pair of sunglasses whilst facing in four different directions, corresponding to the four corners of the screen. Each photograph used the same actor, and the stimuli for each condition differed only on the colour of the sunglasses used. The cue-agent was centrally presented and appeared to gaze at one of four probe stimuli (Figure 1). The probe stimuli consisted of four letters, one in each corner of the screen. These could be either E, U, O, P, S, F, H, L, or A and measured 1.8 × 1.8 cm. The probe stimuli appeared 5 cm away from the initial fixation point.

### 2.4. Procedure

Participants were positioned 57 cm away from the display with their heads in a chinrest. Each participant completed three blocks of 80 trials, completing 240 trials in total. The study had 20% valid trials, 60% invalid trials, and 20% catch trials, in which no change occurred. As there were four potential stimulus locations, a 4:1 ratio of valid to invalid trials was necessary to ensure that the gaze cue was non-predictive of change location.

Prior to starting the main experiment participants were shown two brief videos, each approximately 15 s in length (Video S1: GlassesDemo.wmv). The videos were designed to instil the concept that the actor could ‘see’ whilst wearing one of the pairs of coloured sunglasses and could not ‘see’ whilst wearing the other. The videos featured the actor reaching for and identifying objects. Whilst wearing the ‘seeing’ sunglasses, the actor accurately reached for and identified an object placed in front of them. Whilst wearing the ‘non-seeing’ sunglasses, the actor took time to search for and identify the item placed in front of them, demonstrating that the actor was unable to ‘see’ the item. The use of the videos allowed the participant to form an association of the actor’s behaviour with the type of glasses they were wearing, allowing an immediate attribution to be made upon seeing the actor in the experiment. It was emphasised that the direction in which the actor faced was non-predictive and would not indicate where the change would occur. Further, during the experiment participants were not asked to recall, and neither were they reminded of, whether the agent could ‘see’. 

Trials began with the onset of a fixation point, which was present for 1000 ms. This was replaced with the stimulus array containing four letters, each placed in one of the four corners of the screen for 500 ms. The cue was present for either 100 ms or 1000 ms. The display was then masked for 80 ms, after which the screen refreshed to a new display of the stimulus head and four symbols (Figure 1).

The participant was then required to indicate, via specific buttons on the computer keyboard, whether any of the four symbols had changed. The buttons used were ‘B’ and ‘N’; the participant pressed ‘B’ if they believed one of the symbols had changed and ‘N’ if they believed none had changed.

## 3. Results

Four participants had a high rate of reporting false positives (>35%) on the catch trials and were excluded from the final analysis, leaving a final sample of 30 participants. A 2 × 2 × 2 repeated measures ANOVA, with factors of condition (seeing/non-seeing), SOA (short/long), and validity (valid/invalid) on the probability of correctly identifying a change revealed a main effect of condition (*F*(1,29) = 7.553, *p* = 0.010, *ηρ²* = 0.207), as the proportion of correct responses was greater for seeing trials; a main effect of SOA (*F*(1,29) = 37.953, *p* < 0.001, *ηρ²* = 0.567), as the proportion of correct responses was greater for the long SOA; and a main effect of validity (*F*(1,29) = 5.371, *p* = 0.028, *ηρ²* = 0.156), as the proportion of correct responses was greater for the valid trials. Importantly for the study hypothesis, there was a significant validity × condition interaction (*F*(1,29) = 5.143, *p* = 0.031, *ηρ²* = 0.151). There was no SOA × validity × condition interaction, (F(1,29) = 0.630, *p* = 0.43, *ηρ²* = 0.021) indicating that the nature of the validity × condition interaction did not differ between the short/long SOA.

**Figure 2:**
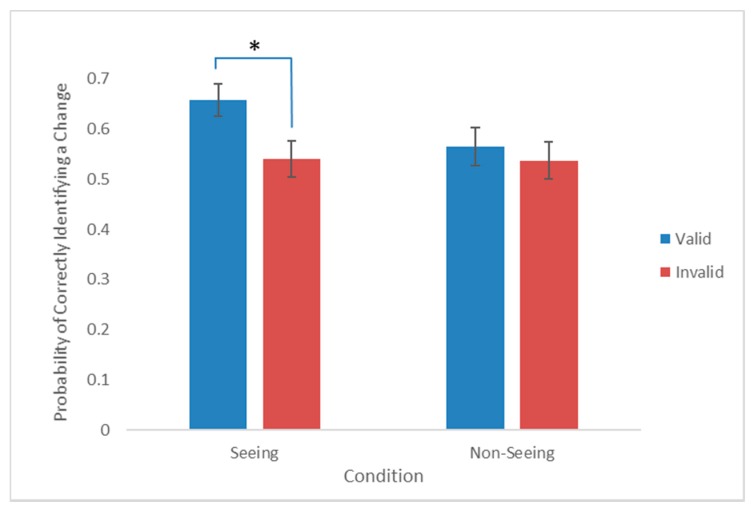
The probability of correctly identifying a change on the valid and invalid trials in the seeing and non-seeing conditions. Error bars show +/−1 within-subject standard error of the mean (S.E.M). * Indicates *p* < 0.05.

The significant validity × condition interaction was analysed using paired samples *t*-tests. The analysis revealed that participants were significantly more likely to detect a change in the seeing condition when the cue was valid (M = 0.66, SD = 0.18), compared to when the cue was invalid (M = 0.54, SD = 0.20; t(29) = 3.002, *p* = 0.005, *d_z_* = 0.55). By contrast, in the non-seeing condition participants did not display this cueing effect and there was no significant difference between the valid (M = 0.57, SD = 0.21) and invalid trials (M = 0.54, SD = 0.20; t(29) = 0.903, *p* = 0.374, *d_z_* = 0.17). Taken together, these results demonstrate that participants were influenced by the mental state of the cue-agent as when the cue-agent could see, validly cued targets were more likely to be detected than invalidly cued targets. However, when the cue-agent could not see, validly cued targets were no more likely to be detected than invalidly cued targets (Figure 2).

## 4. Discussion

In the current study, we used a gaze cueing paradigm within a change detection task to investigate to what extent the modulation of gaze-cueing by mental state attributions observed by Teufel et al. [2] generalised to other tasks. The results demonstrated that participants were significantly more likely to correctly detect a change when the cue was valid, and they believed the cue-agent to be able to see. This therefore confirms that participants were affected by the perceived mental state of the cue-agent, and that this modulated the gaze cueing effect present in the data. Critically, this study investigated if a mental state attribution could mediate participants’ performance on a change detection task. Our results offer an important extension of previous studies, demonstrating that a mental state attribution can directly affect our perception of visual stimuli, in this case reducing participants’ susceptibility to change blindness by heightening their awareness of locations within the visual array. 

Of key importance, our participants showed the effect of a mental state attribution when seeing and non-seeing trials were randomly interleaved, thus ruling out the possibility that change in gaze cueing were caused by the adoption of an attentional set across a block of trials. As participants were never directly asked to register the mental state of the agent, we can also confidently exclude the suggestion that reduced gaze cueing in the ‘non-seeing’ condition was due to demand characteristics. Further, whilst Teufel et al. argued that MSAs automatically mediated reflexive gaze cueing, the experimental paradigm used within their study relied upon the explicit application of a mental state. During the current study, participants were never explicitly asked to consider the perspective of the agent and yet still showed the effect of a mental state attribution, suggesting that participants implicitly attributed a mental state to the cue-agent. These results are therefore consistent with those of Teufel and colleagues [2] and additionally indicate that MSAs can occur implicitly, without conscious awareness, and yet still modulate social attention. 

On first inspection, the results from this study are not consistent with recent research claiming that mental states attributions do not influence attentional orienting [11]. However, this difference may arise from the nature of the mental state manipulation used within each paradigm. Line-of-sight manipulations, such as the use of barriers [11,12,14], rely upon alterations within the stimulus display in order to alter the perspective of the cue-agent. This may be a critical difference, as manipulating the cue-agent rather than the environment may make it easier for mental state attributions to modulate gaze cueing. Specifically, when mental states are changed by altering the cue-agent, the information about gaze direction and mental state are available at the same location, which is the location the participant is fixating. In this case, gaze direction and mental state may be computed simultaneously and thus have greater opportunity to interact. However, when mental states are changed by altering the environment, the relevant information is spatially separate, such that gaze direction is available at fixation, but information about whether the line of sight is open or occluded is in the periphery. This manipulation arguably adds an extra level of complexity to the paradigm [23]. In this case, participants might process the centrally presented gaze cue first and then compute the mental state. Given the speed of reflexive gaze cueing, it may be that attention is oriented before it can be modulated by mental state attributions. We therefore tentatively propose that MSA can influence attentional orienting when information about the mental state and the gaze cue are found at the same location. 

One other key difference between this and previous studies is the use of videos prior to the main experiment. The videos were designed to instill the knowledge of when the actor could or could not see and extend the usual use of written or verbal instructions [2]. Arguably the videos allowing the participants prior experience of the actor’s behaviour (in a real-life setting) acted to reinforce the theory of mind processes drawn upon when completing the experimental task, therefore promoting the effects found within this study. Therefore, a combination of the use of a more sensitive task and more direct mental state manipulation may have led to the perspective taking effects found within the current study. 

## 5. Conclusions

This study investigated whether the attribution of a mental state to a cue-agent mediated attentional orienting within a change detection task. Our results clearly supported our predictions, with participants significantly more likely to correctly identify if a change had occurred on valid trials when they believed that the cue-agent was able to ‘see’. The computation of the cue-agent’s mental state thus directly impacted participants’ performance on a perceptual task, demonstrating that the attribution of mental states mediates the gaze cueing effect and can influence the perception of visual information around us.

## Supplementary Materials

The following are available online at http://www.mdpi.com/2411-5150/2/1/11/s1, Video S1: GlassesDemo.wmv.
